# Supplementary figures and images for: Association between Preoperative Nutritional Status and Clinical Outcomes of Patients with Coronary Artery Disease Undergoing Percutaneous Coronary Intervention
Source: Nutrients. 2020 May 2;12(5):1295. doi: 10.3390/nu12051295 (PMC7282248; doi:10.3390/nu12051295)

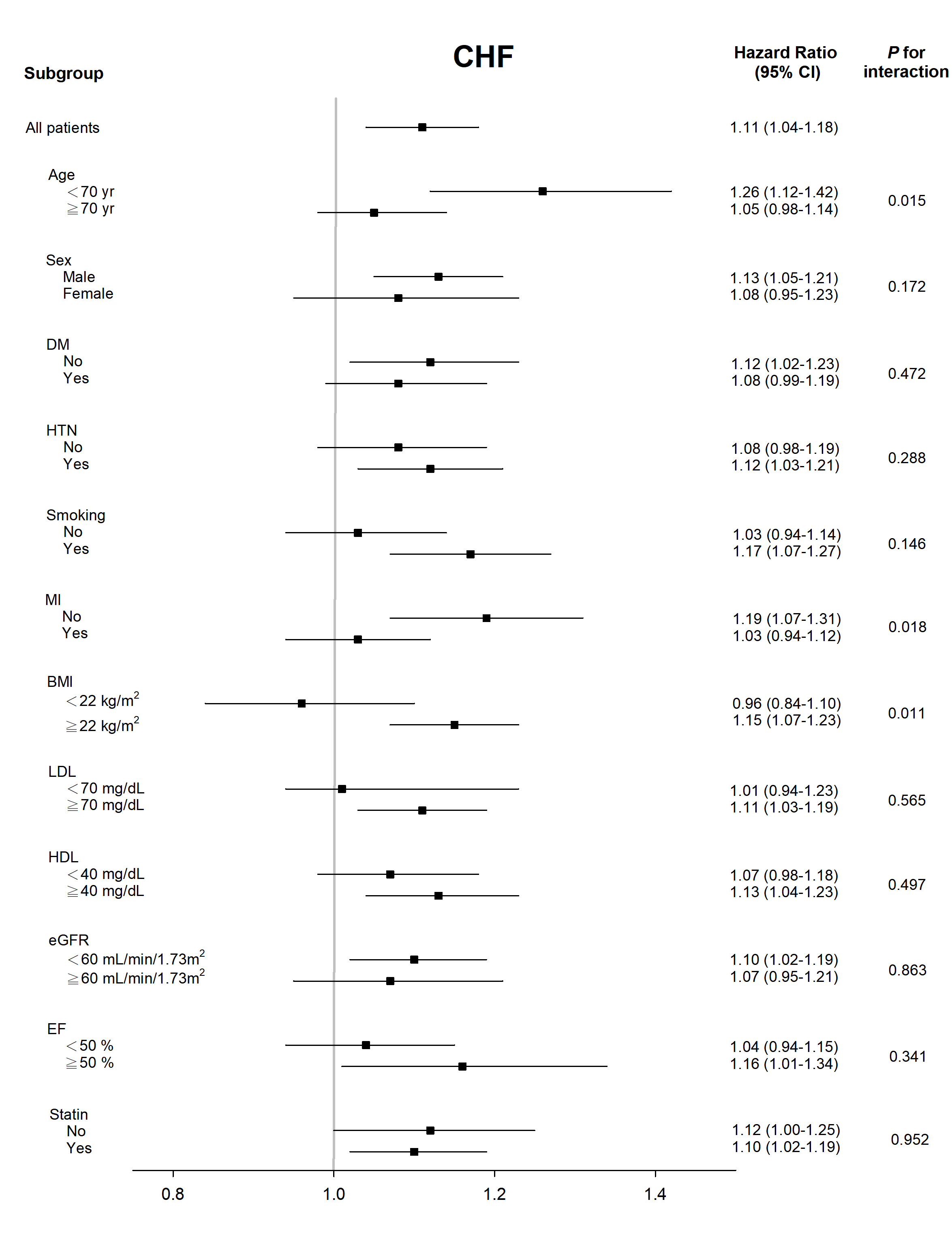

Supplement: Supplementary file 1 [file nutrients-12-01295-s001.zip › nutrients-781730-supplementary/Supplement figures 1-5/Supp Figure 1_Forest plot of hazard ratio for CHF.JPG]

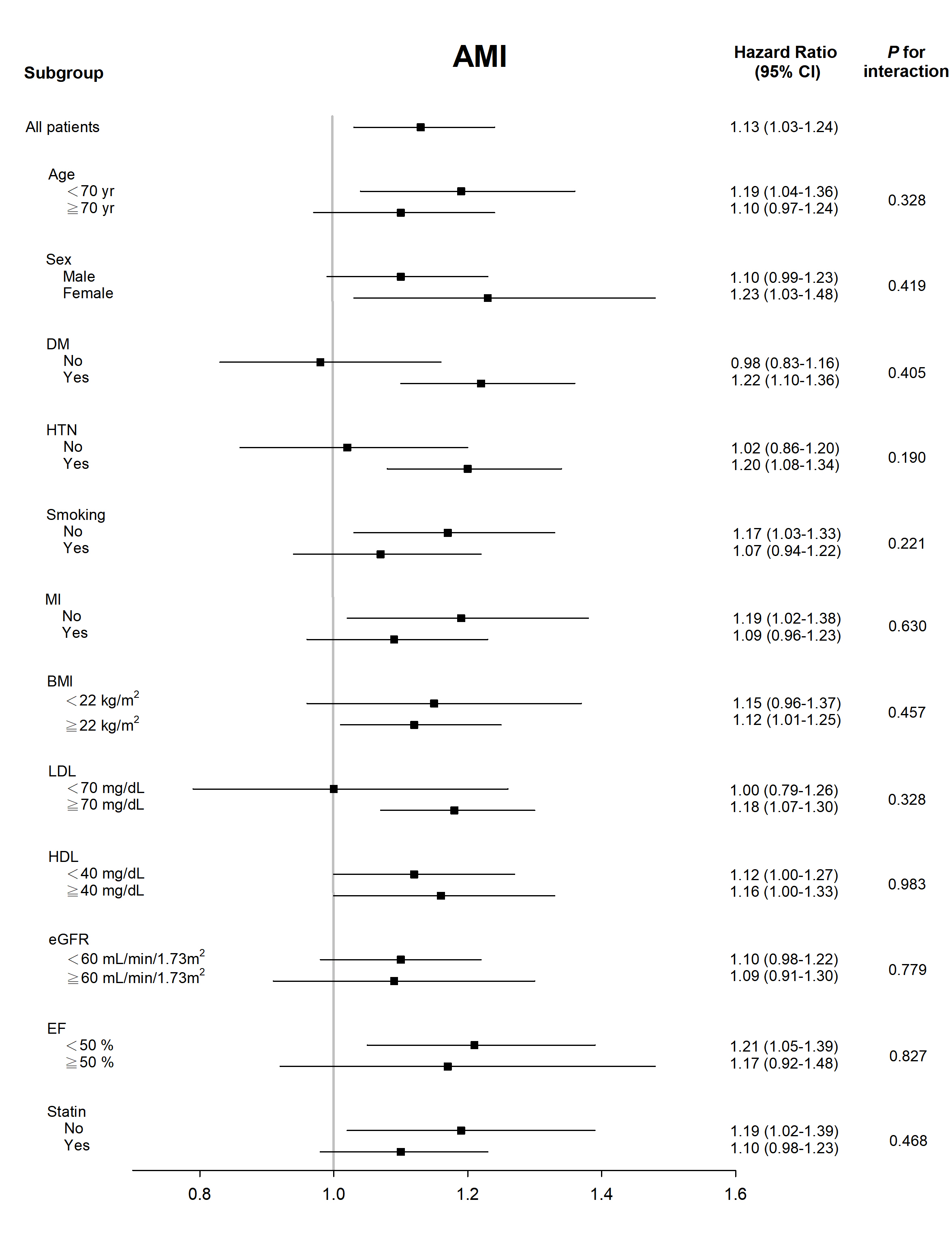

Supplement: Supplementary file 1 [file nutrients-12-01295-s001.zip › nutrients-781730-supplementary/Supplement figures 1-5/Supp Figure 2_Forest plot of hazard ratio for AMI.JPG]

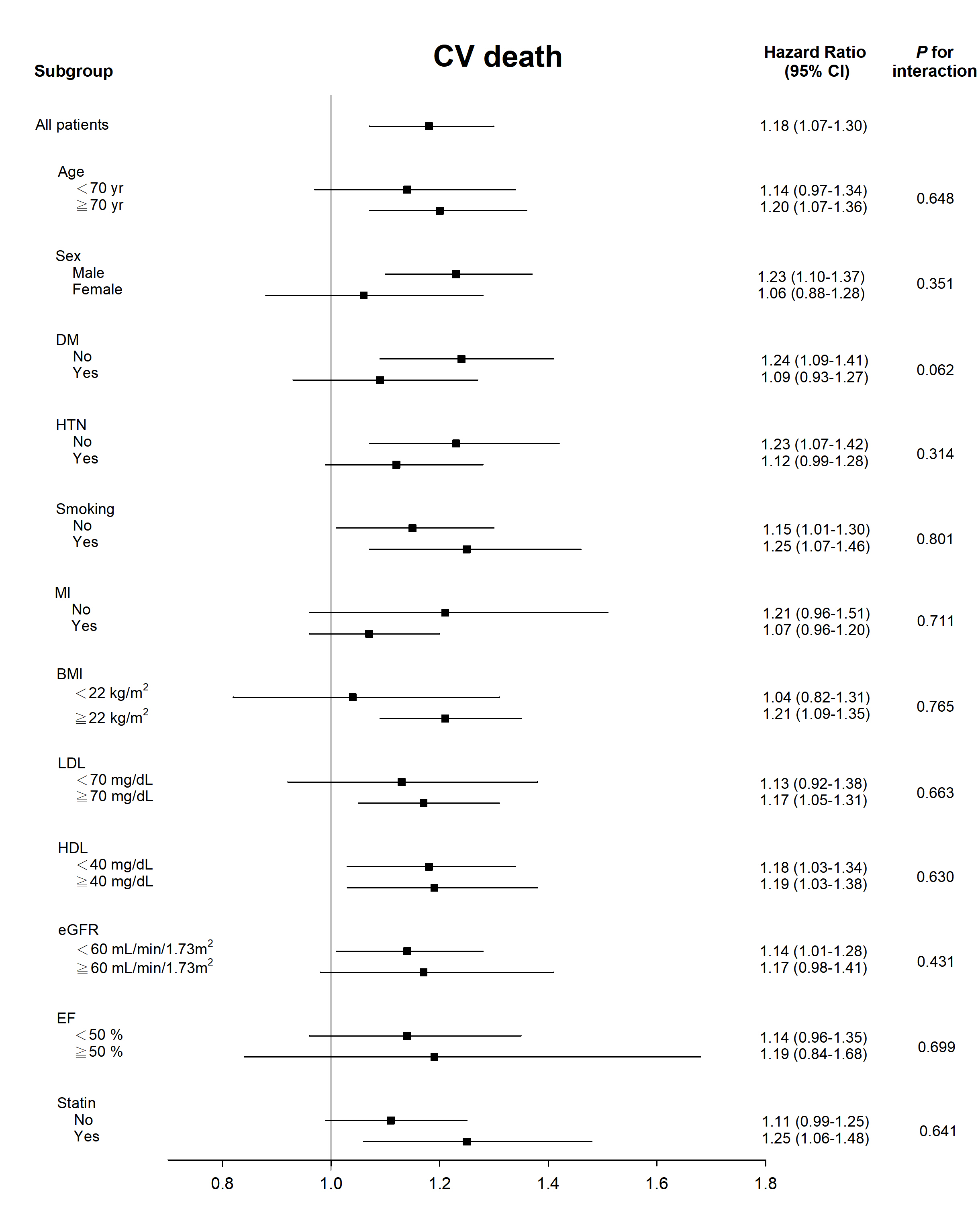

Supplement: Supplementary file 1 [file nutrients-12-01295-s001.zip › nutrients-781730-supplementary/Supplement figures 1-5/Supp Figure 3_Forest plot of hazard ratio for CV Death.JPG]

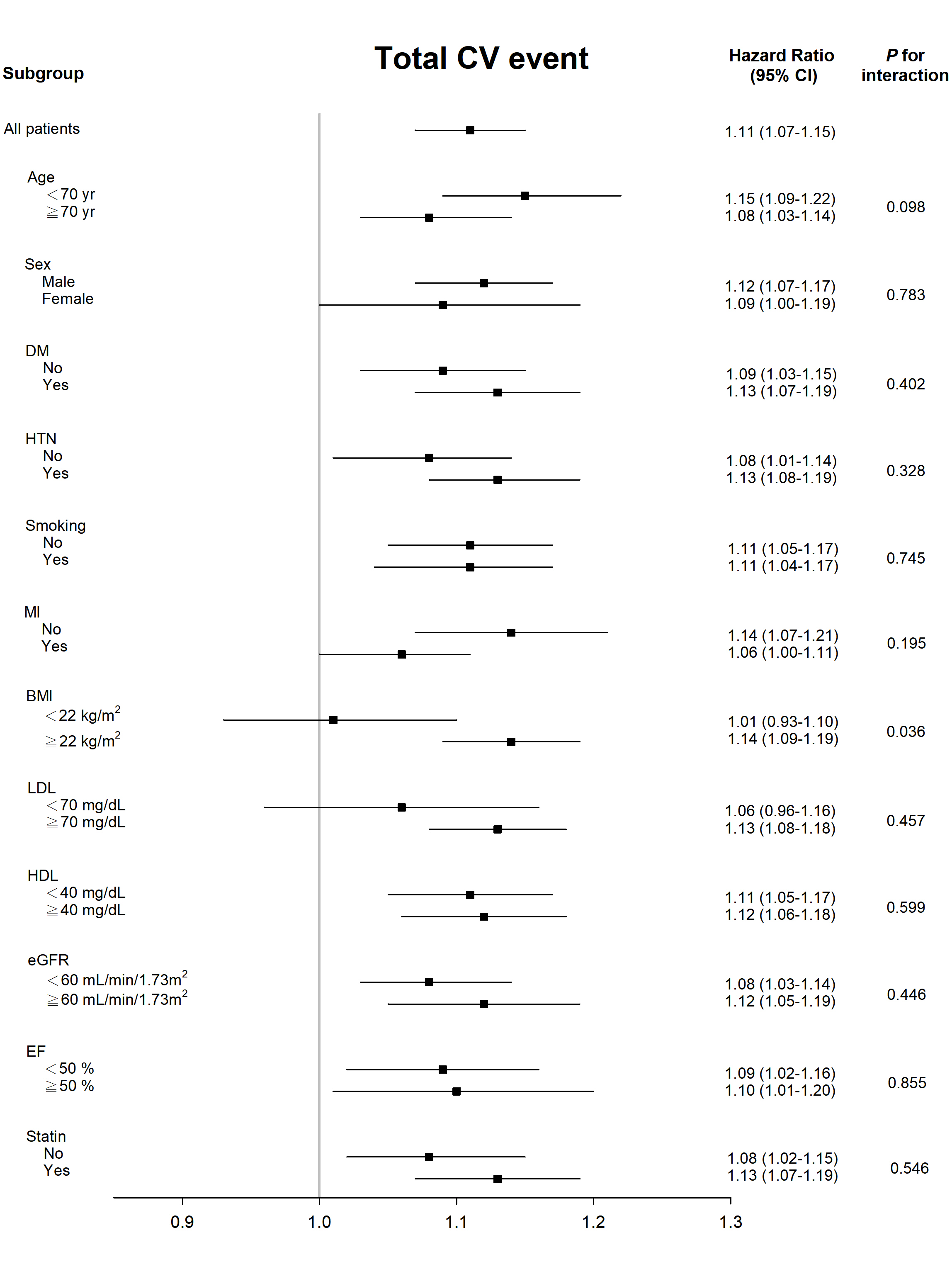

Supplement: Supplementary file 1 [file nutrients-12-01295-s001.zip › nutrients-781730-supplementary/Supplement figures 1-5/Supp Figure 4_Forest plot of hazard ratio for Total CV Event.JPG]

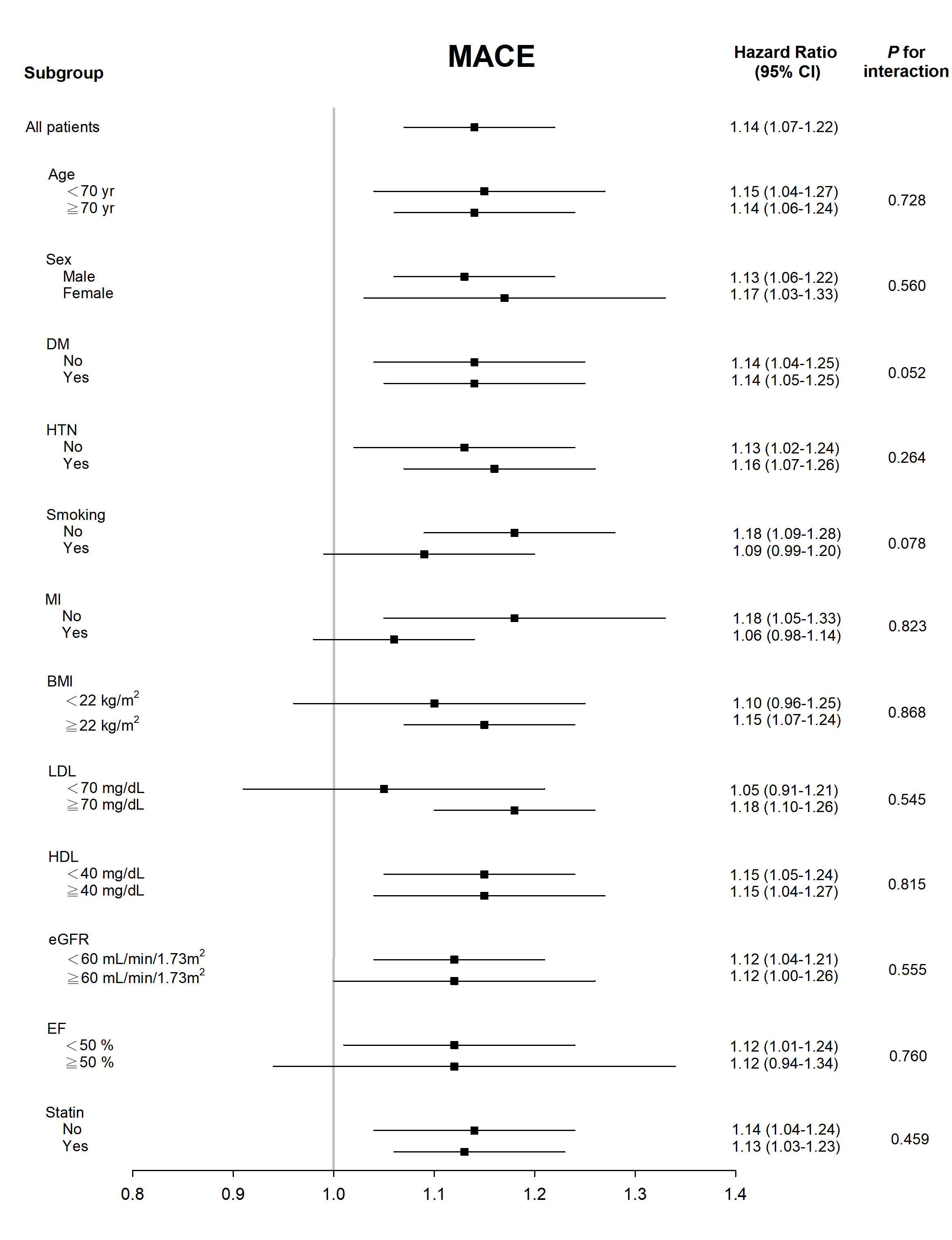

Supplement: Supplementary file 1 [file nutrients-12-01295-s001.zip › nutrients-781730-supplementary/Supplement figures 1-5/Supp Figure 5_Forest plot of hazard ratio for MACE.JPG]
